# Exploratory Urinary Proteomic Profiling in Pregnancies with Fetal Aneuploidies: Molecular Insights into Maternal–Fetal Metabolic Communication

**DOI:** 10.3390/cimb47120973

**Published:** 2025-11-24

**Authors:** Răzvan Lucian Jurca, Maria-Andreea Soporan, Ioana-Ecaterina Pralea, Ioana Gheorghiu, Iulia Rus, Florin Stamatian, Cristina-Adela Iuga

**Affiliations:** 1 Mother and Child Department, Obstetrics and Gynecology I, “Iuliu Hațieganu” University of Medicine and Pharmacy Cluj-Napoca, 400347 Cluj-Napoca, Romania; 2 Personalized Medicine and Rare Diseases Department, MEDFUTURE—Institute for Biomedical Research “Iuliu Hațieganu” University of Medicine and Pharmacy, Louis Pasteur Street 6, 400349 Cluj-Napoca, Romania; 3 Department of Pharmaceutical Analysis, Faculty of Pharmacy, “Iuliu Hațieganu” University of Medicine and Pharmacy, Louis Pasteur Street 6, 400349 Cluj-Napoca, Romania; 4 Department of Medical Informatics and Biostatistics, “Iuliu Hațieganu” University of Medicine and Pharmacy, Louis Pasteur Street 6, 400349 Cluj-Napoca, Romania; 5 Department of Clinical Pharmacy, Faculty of Pharmacy, “Iuliu Hațieganu” University of Medicine and Pharmacy, Louis Pasteur Street 6, 400349 Cluj-Napoca, Romania; 6 Imogen Clinical Research Centre, 400347 Cluj-Napoca, Romania

**Keywords:** mass spectrometry, label-free proteomics, urinary proteomic profiling, fetal aneuploidy, maternal–fetal communication

## Abstract

Maternal–fetal communication involves complex molecular exchanges that can be perturbed by chromosomal abnormalities. Despite the growing use of omics approaches in prenatal research, maternal urine remains underexplored as a non-invasive source of molecular information reflecting both systemic and pregnancy-specific processes. This study performed an exploratory, label-free mass spectrometry-based proteomic analysis of maternal urine from pregnancies with normal karyotype (*n* = 15) and those affected by fetal aneuploidy (*n* = 9; trisomy 21, 18, 13, or monosomy X). A total of 861 proteins were identified across all samples, of which 42 significantly differed between groups (*p* ≤ 0.05, |fold change| ≥ 1.2). Ten proteins, including TFF1, TFF3, KRT76, CD300, PVR, VWA1, FBLN1, FGA, AZGP1, and MAT1, were more abundant in aneuploid pregnancies, suggesting roles in immune modulation, epithelial restitution, metabolic control, and extracellular matrix remodeling. Conversely, 32 proteins with lower abundance were primarily involved in immune regulation, structural organization, and energy metabolism, consistent with impaired placental and vascular adaptation. These findings reveal distinct urinary proteomic signatures associated with fetal aneuploidy, highlighting biologically relevant pathways that may advance understanding of maternal–fetal metabolic communication.

## 1. Introduction

Pregnancy induces profound immunological and metabolic adaptations at the maternal–fetal interface. Fetal chromosomal abnormalities such as trisomies 21, 18, and 13 are known to disrupt these tightly regulated processes affecting placental development, immune regulation, and maternal metabolic balance [1]. Several investigations have shown that aneuploidy alters placental structure and function, resulting in impaired placental maturation, increased vascular resistance, and higher rates of fetal vascular malperfusion [2,3,4]. In trisomy 21, particularly, placentas often exhibit structural immaturity and heightened oxidative stress, compromising their architecture and functionality.

Mass spectrometry (MS)-based omics technologies have become essential in both research and clinical practice. Beyond the research setting, MS already has an established role in newborn screening where it enables the sensitive detection of inborn errors of metabolism, highlighting its translational potential [5]. In proteomics, MS-based workflows enable global, quantitative profiling of proteins and post-translational modifications, offering valuable insights into maternal–fetal molecular communication. Several studies have successfully used proteomics to explore pregnancy-related conditions, including preeclampsia, gestational diabetes, and placental dysfunction [6,7]. While most prenatal proteomic studies have focused on amniotic fluid, serum, and placental tissues, maternal urine remains an unexplored yet highly promising biological matrix for non-invasive biomarker discovery. Also, urine can be collected non-invasively in large volumes and can reflect both systemic and pregnancy-specific physiological states [8,9]. On this note, urinary proteomics has revealed dynamic molecular signatures of gestational adaptation, including proteins involved in metabolic regulation, immune responses, and extracellular matrix remodeling [9,10,11]. Furthermore, urinary proteomic and peptidome profiling was used for identifying candidate biomarkers for fetal aneuploidy supporting the potential of urine-based non-invasive prenatal screening [12,13,14]. Recent MS-based work has shown that maternal urine proteomic and metabolomic profiles can capture disturbances in lipid and amino acid metabolism, gluconeogenesis, and immune pathways associated with aneuploid pregnancies [1,12,15].

Despite these advances, comprehensive characterization of the maternal urinary proteome in pregnancies affected by fetal aneuploidy remains limited.

Building on previous evidence linking chromosomal abnormalities to altered placental, immune, and metabolic function [1], the present study was designed to determine whether pregnancies complicated by fetal aneuploidy exhibit distinctive maternal urinary proteomic patterns compared to euploid pregnancies. Such differences, reflecting disrupted maternal–fetal metabolic and immune signaling, could reveal potential non-invasive molecular indicators of fetal chromosomal abnormalities and provide candidate biomarkers for future screening applications. In this context, the present study performs an exploratory, label-free, mass-spectrometry-based proteomic profiling of maternal urine collected from pregnancies with normal karyotype and those affected by fetal aneuploidy (trisomy 21, 18, 13, or monosomy X).

## 2. Materials and Methods

### 2.1. Participants, Demographic Data and Eligibility Criteria

A case–control study was performed between July 2023 and May 2024 at the IMOGEN Research Institute, Cluj County Emergency Clinical Hospital. Twenty-four pregnant women in their first and second trimesters were enrolled after providing written informed consent as part of a research program aimed at exploring novel non-invasive urinary markers for trisomy 21 (Down syndrome). The study was approved by the Scientific Research Ethics Committee from “Iuliu Hațieganu” University of Medicine and Pharmacy Cluj-Napoca (approval no.253/14 July 2020) and was carried out in accordance with the principles of the Declaration of Helsinki. Amond the participants, 9 were carrying fetuses with confirmed chromosomal abnormalities, namely, trisomy 21 (Down syndrome, n = 4), trisomy 13 (Patau syndrome, n = 2), trisomy 18 (Edwards syndrome, n = 1) and a monosomy (Turner syndrome, n = 3), and fifteen had fetuses with normal karyotypes serving as controls. Prenatal diagnosis of aneuploidies involving chromosomes 13, 18, 21, X and Y was performed on DNA extracted from amniotic fluid. Initial detection was carried out using quantitative fluorescent PCR (QF-PCR), and all positive cases were subsequently confirmed by standard karyotyping.

Participants were eligible for inclusion if they had a viable pregnancy with a gestational age between 12 and 18 weeks. Gestational age was determined using standard clinical criteria, including a reliable last menstrual period, documented dates of negative and positive pregnancy tests, first-trimester ultrasound findings, and pre-amniocentesis fetal biometric measurements. Exclusion criteria included incomplete medical records, absence of signed informed consent, or insufficient sample quality for analysis. One urine sample was excluded from the study because no detectable protein content was recovered.

Demographic characteristics, relevant medical history, and pregnancy-related data (gestational age, complications) were recorded at the time of enrolment (Table 1). Data was analyzed using descriptive and inferential statistics. Statistical analyses were conducted in STATISTICA (Version 13.5, StatSoft, OK, USA), and Jamovi (Version 2.6, Sydney, Australia). We described the categorial variables as absolute and relative frequencies and percentages (n, %), continuous variables (maternal age, gestational age) were expressed as means ± standard deviations and compared between case and control groups using independent samples *t*-tests (Welch’s correction applied where variances were unequal). Associations between categorical variables and group assignments were tested using Chi-square tests of independence. Fisher’s exact test was applied for 2 × 2 contingency tables or where expected cell counts were below 5. All statistical tests we performed were two-sided tests; a significant result being considered for a *p*-value < 0.05.

### 2.2. Urine Sample Collection

Urine samples were collected by participants using the midstream clean-catch method with approximately 20 mL obtained from either the first or second morning void. Following collection, samples were transported to the laboratory within two hours, under controlled temperature conditions. From each urine sample, 5 mL were aliquoted for routine urine analysis using a BC400 urine analyzer (Contec Medical Systems Co., Ltd., Qinhuangdao, China). The remaining urine was centrifuged at 10,000× *g* for 10 min to remove debris and cellular material. The clarified supernatant was transferred into low-retention 1.5 mL microcentrifuge tubes and stored at −80 °C until subsequent protein extraction and mass spectrometry analysis.

### 2.3. Sample Preparation for MS-Based Proteomics

For each sample, 900 µL urine were concentrated to a final volume of 100 µL using Amicon^®^ Ultra-0.5 Centrifugal Filter Units (Millipore-Sigma, Burlington, MA, USA) with a 3kDa molecular weight cutoff. Proteins were extracted using the methanol–chloroform (MeOH-Chl) precipitation method, the protein pellet being further resuspended in 8 M urea prepared in 50 mM Tris-HCl buffer (UTris, pH 8.0). Protein extraction was enhanced by sonication (3 × 3 s, 90% power—Bandelin Electronic GmbH, Berlin, Germany). After centrifugation, the supernatant was used to determine the total protein concentration d using the microBradford assay (BioRad Laboratories, Munich, Germany) against a standard curve of bovine serum albumin (BSA). A sample volume equivalent to 2 µg of protein was reduced by 25 mM dithiotretiol (DTT) (60 °C for one hour), followed by alkylation by iodoacetamide (IAA) (final concentration of 40 mM for 30 min at 37 °C). Proteins were digested overnight at 37 °C with proteomics-grade trypsin (MilliporeSigma, Burlington, MA, USA) at an enzyme-to-protein ratio of 1:50. Digestion was halted by acidification and all samples underwent peptide purification using ZipTip µC18 tips (Millipore Sigma, Burlington, MA, USA). The purified peptides were concentrated by vacuum evaporation (Thermo Fisher Scientific, Waltham, MA, USA) and reconstituted in 20 µL of 0.1% formic acid prior to mass spectrometry injection.

### 2.4. Label Free Nano-LC-IMS-MS and Data Analysis

Data acquisition was performed as previously described [16]. Specifically, proteome profiling data acquisition was carried out by injecting 300 ng protein on a reversed-phase Acquity UPLC M-class Symmetry C18 trap column (180 µm × 20 mm, 5 µm particle size, Waters Corp., Milford, MA, USA) at a flow rate of 5 µL/min in 99% solvent A (0.1% formic acid in water). After a two-minute loading and washing, peptides were separated on nanoAcquity UPLC M-Class T3 reversed-phase column (75 µm × 150 mm, 1.8 µm particle size, Wexford, Ireland) and eluted at a flow rate of 0.3 µL/min using a 45-min multistep concave gradient ranging from 5 to 85% solvent B (0.1% formic acid in acetonitrile). Column temperature was set at 55 °C. Eluted peptides were ionized in positive mode using the nanoESI source and analyzed on a SYNAPT G2-Si HDMS instrument (Waters Corporation, Wilmslow, UK) operated in high-definition ion mobility mode (HDMSe). Data were acquired in resolution mode over the 50–2000 *m*/*z* mass range with a scan time rate of 0.5 s. The following source and analyzer settings were applied: capillary voltage was 2.5 kV, source temperature 80 °C, sampling cone 40 V, and cone gas flow 30 L/h. LC–MS data were acquired in data-independent acquisition (DIA) mode using the ion mobility enabled HDMSE mode. For IMS separation, a traveling-wave height of 40 V was applied, and the wave velocity was linearly ramped from 800 to 500 m/s across the full IMS cycle. Wave velocities in the trap and transfer cells were 311 m/s and 190 m/s, respectively, with wave heights of 4 V in both regions. Spectra were collected in resolution mode over the *m*/*z* range 50–2000, with a scan time of 0.5 s. For low-energy scans, the collision energy was fixed at 4 eV (trap) and 2 eV (transfer), while in high-energy scans, the transfer collision energy was ramped from 19 to 45 eV. Post-acquisition, lock mass correction was applied using the doubly charged monoisotopic ion of [Glu1]-Fibrinopeptide B (*m*/*z* 785.8426).

Comparative proteomic analysis was carried out using Progenesis QIP v.4.2 (Nonlinear Dynamics, Waters Corporation). A target-decoy UniProtKB/Swiss-Prot Human database (20,361 entries, downloaded January 2022) was used for protein identification with the following parameters: trypsin as the digestion enzyme, allowing a maximum of one missed cleavage; fixed modification: fixed modification—carbamidomethylation (C); variable modification—methionine oxidation (M); false discovery rate (FDR) < 1%; and peptide precursor mass error tolerance of 10 ppm. Database searching was performed using trypsin as the digestion enzyme, allowing a maximum of one missed cleavage. This threshold was selected based on digestion efficiency metrics from Progenesis QI for Proteomics, which showed >95% of peptides were fully cleaved or contained a single missed cleavage. Ion matching criteria were set as follows: (i) minimum of one fragment ion match per peptide ion; (ii) at least three fragment ions matched per protein; and (iii) at least one unique peptide match per protein. Data normalization was performed within Progenesis QIP v.4.2 using the “Normalize to all proteins” option. Quantification was based on non-conflicting peptides, and resulting normalized data were exported as .csv files for downstream statistical analysis.

Post-processing of normalized quantitative data was performed using MetaboAnalyst 6.0 (https://www.metaboanalyst.ca, accessed on 8 September 2025). Proteins with more than 30% missing values were removed and remaining missing values were imputed using k-nearest neighbors based on similar features (KNN, feature-wise option). No further filtering was applied, and data were log10-transformed. Differential expression analysis was performed using a two-sample *t*-test with unequal group variance, with significance defined as *p* ≤ 0.05 and absolute fold change |FC| ≥ 1.2. Data visualizations, including volcano plots, PCA plots, heatmaps, and hierarchical clustering, were carried out using the same tool. Protein identifiers were standardized according to gene nomenclature. Functional annotation and classification of differentially abundant proteins were carried out using the PANTHER database (version 19) (https://pantherdb.org, accessed on 30 July 2025).

## 3. Results

### 3.1. Clinical and Demographic Profile

A total of 24 urine samples from pregnant women were analyzed, including nine samples with confirmed fetal aneuploidy (case group) and fifteen samples from euploid pregnancies (control group). Table 1 summarizes the Group differences in maternal and pregnancy characteristics. Maternal age was significantly higher in the case group compared to controls (Mann–Whitney U test, *p* = 0.007). Similarly, gestational age at inclusion was significantly higher in the case group (Mann–Whitney U test, *p* < 0.001).

Regarding lifestyle factors, a significantly larger proportion of women in the case group reported active smoking compared with the control group (Fisher’s exact test, *p* = 0.047). Folic acid supplementation patterns did not differ significantly between groups, with nearly all participants in both cohorts reporting (Fisher’s exact test, *p* = 0.375,).

No statistically significant differences were found between groups regarding marital status (Fisher’s exact test, *p* = 0.356), place of origin (Fisher’s exact test, *p* = 1.0), educational level (Fisher’s exact test, *p* = 0.678), dysmenorrhea (Fisher’s exact test, *p* = 0.40), or threatened miscarriage (Fisher’s exact test, *p* = 1.0).

### 3.2. Protein Identification and Functional Annotation

Comparative analysis in Progenesis QI for Proteomics identified 861 urinary proteins across all 24 maternal samples, using a joint alignment strategy to establish a consistent protein identification framework. This approach enabled reliable downstream comparison of protein abundance between the aneuploid (n = 9) and euploid (n = 15) groups. The complete list of identified and quantified proteins, along with their distribution across individual samples is provided in Table S1.

Functional annotation of the identified urinary proteome is summarized in Figure S1. Gene Ontology (GO) classification confirmed that the dataset broadly represented expected molecular categories, including binding and catalytic activities, as well as proteins involved in cellular and metabolic processes.

Beyond these general functions, PANTHER protein class analysis highlighted an enrichment of defense/immunity proteins (PC00090, 13.9%), metabolite interconversion enzymes (PC00262, 13.0%), and protein-modifying enzymes (PC00260, 10.0%). Several functional groups underscored the immunologically active state of pregnancy, including proteins related to response to stimulus (GO:0050896, 22.7%) and immune system processes (GO:0002376, 12.3%). Proteins associated with developmental (GO:0032502, 10.0%) and multicellular organismal processes (GO:0032501, 9.5%) were also prominently represented.

Additional categories of interest included antioxidant activity (GO:0016209, 1.5%) and homeostatic processes (GO:0042592, 2.0%), reflecting oxidative stress and metabolic adaptations, as well as molecular transducer activity (GO:0060089, 3.5%) and transmembrane signal receptors (PC00197, 4.1%). Collectively, these enrichments emphasize the ability of urinary proteomics to capture systemic and pregnancy-specific molecular adaptations, providing a biologically relevant foundation for subsequent comparisons between aneuploid and euploid pregnancies.

### 3.3. Differentially Abundant Proteins (Aneuploidy vs. Control)

Principal component analysis (PCA) was performed using all identified proteins to explore overall data structure (Figure S2). The first five components explained 63.1% of the total variance, with PC1 accounting for 32.9%. While some degree of clustering between cases and controls was observed, a certain overlap was also present, which is consistent with the exploratory nature of this dataset and the biological heterogeneity of the study groups. To further illustrate the data distribution, a 3D PCA plot is provided in Figure S2.

**Figure 1:**
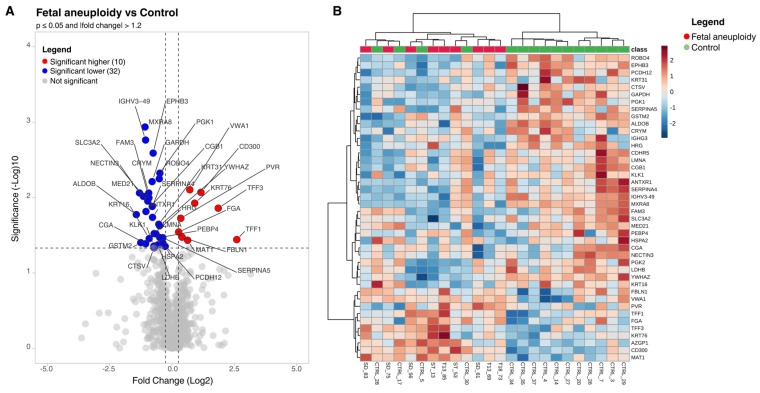
Proteome profile differentiation between study groups: (**A**) Volcano plot showing differential protein expression between fetal aneuploidy and control groups. Proteins with |fold change| ≥ 1.2 and *p* ≤ 0.05 were considered significantly differentially expressed. Red and blue dots indicate proteins with higher and lower abundance, respectively, while gray dots represent non-significant different proteins. (**B**) Heatmap illustrating the expression profiles of the 42 significantly differentially abundant proteins across all urine samples. Expression values were normalized and clustered using hierarchical clustering (distance: Euclidean; linkage: average).

Comparative statistical analysis identified 42 proteins that significantly discriminated between mothers carrying aneuploid fetuses and those with normal karyotypes (*p* ≤ 0.05, |fold change| > 1.2) (Figure 1, Table S2). Hierarchical clustering of the 42 differentially abundant proteins revealed partial separation between aneuploid and control samples, with moderate within-group variability reflecting individual metabolic and physiological differences. Among the differentially abundant proteins, ten proteins owed higher abundance in the aneuploidy group, including markers of epithelial restitution (TFF1, TFF3, KRT76), immune modulation (CD300, PVR), extracellular matrix and vascular regulation (VWA1, FBLN1, FGA) and metabolic control (AZGP1, MAT1). Conversely, 32 proteins showed lower abundance, encompassing immune components, structural proteins, metabolic enzymes, and signaling regulators. Together, these opposing patterns point to widespread perturbations in epithelial integrity, immune surveillance, vascular remodeling, and metabolic adaptation at the maternal–placental interface.

### 3.4. Contextual Comparison with the Reference Urinary Proteome

**Figure 2:**
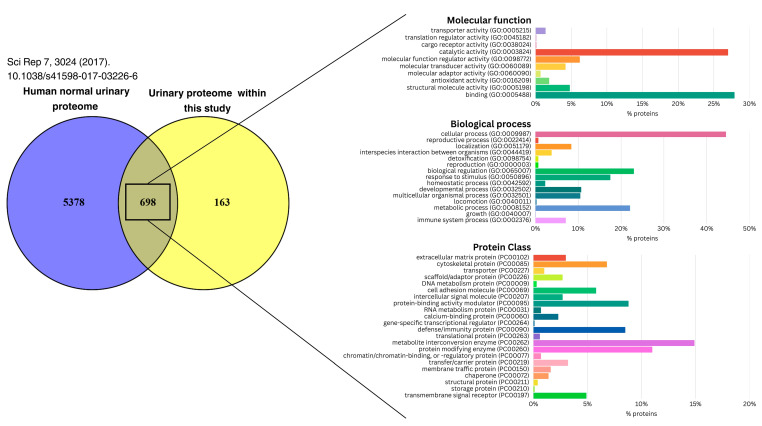
Overlap between the urinary proteome identified in this study and the normal human urinary proteome reported by Zhao et al. [17]. (**Left panel**): Venn diagram illustrating the shared and unique proteins. (**Right panel**): Functional annotation of the proteins common to both datasets.

To contextualize our findings, we compared the urinary proteome obtained in this study (861 proteins) with the comprehensive reference proteome of normal human urine reported by Zhao et al. [17]. When all 24 samples were analyzed jointly, 698 proteins (81.1%) overlapped with the reference dataset, whereas 163 proteins (18.9%) were uniquely detected in this study (Figure 2, Table S3).

Functional annotation of the overlapping proteins showed predominant binding (27.9%) and catalytic (27.0%) activities, mainly associated with cellular processes (44.5%), biological regulation (23.0%), metabolic processes (22.1%), response to stimulus (17.5%), and developmental processes (10.7%).

In contrast, the 163 uniquely detected proteins (Figure S3, Table S3) exhibited enrichment in defense/immunity proteins (37.1%), cell adhesion molecules (6.3%), protein-modifying enzymes (5.7%), protein-binding activity modulators (4.4%), and cytoskeletal proteins (3.8%). Further interrogation of the Human Urinary Proteome Database for functional associations revealed that nine of these proteins—SERPINA5, PLG, COL18A1, FOLR1, KLK11, MSLN, MMP7, GSTM5, and RNASE2—have previously been implicated in diverse disease processes, supporting the biological and potential clinical relevance of the uniquely detected urinary proteins in this study.

## 4. Discussion

Pregnancy involves finely tuned immune–metabolic adaptations that sustain maternal–fetal equilibrium. Disruption of these processes by fetal aneuploidy can alter placental maturation, vascular remodeling, and oxidative balance, ultimately compromising fetal development [1,4,18]. In this study, we applied a label-free, mass spectrometry-based proteomic approach to maternal urine to capture the systemic molecular signatures associated with fetal aneuploidy. This non-invasive matrix provides a unique window into maternal–fetal metabolic communication, complementing insights gained from serum, amniotic fluid, and placental analyses [1,15].

**Sample size rationale.** The cohort size was primarily determined by feasibility and the availability of clinically confirmed aneuploid pregnancies within the 11-month recruitment period, reflecting the naturally low prevalence of these conditions. The study was therefore designed as an exploratory, hypothesis-generating analysis aimed at capturing preliminary proteomic trends in maternal urine. Our findings revealed 42 differentially abundant proteins (Table S3) that highlight converging pathways of immune regulation, epithelial and extracellular matrix remodeling, and metabolic adaptation, suggesting coordinated disturbances in maternal–fetal homeostasis. These are further categorized according to their molecular functions and biological processes, allowing the delineation of shared mechanisms and pathway-level alterations characterizing aneuploid pregnancies.

**Epithelial, Placental Barrier and Structural Proteins**. A central observation of this study was the increased urinary abundance of epithelial and structural proteins in aneuploid pregnancies, particularly trefoil factors (TFF1, TFF3) and keratin 76 (KRT76). These proteins support epithelial restitution, mucosal protection, and barrier integrity. Importantly, TFF1 and TFF3, both encoded on chromosome 21, were markedly upregulated in the aneuploid group, consistent with enhanced epithelial stress responses and immune modulation. Although reports confirm elevated serum TFF levels during pregnancy, they have not been validated as specific serum biomarkers for trisomy 21 [19]. Among the trefoil family, TFF3 is the predominant isoform in urine while TFF1 and TFF2 occur at lower levels. Increased urinary levels of TFF2 and TFF3 have been associated with inflammation and neoplastic processes [20]. Similarly, elevated levels of TFF3 in amniotic fluid from Down syndrome pregnancies have been interpreted as markers of epithelial stress and inflammation rather than direct aneuploidy-driven regulation [21].

Keratins, particularly KRT7, were also found at higher abundance in aneuploid pregnancies within this cohort. Beyond their structural roles in epithelial tissues, keratins contribute to trophoblast function, placental development, and fetal growth [22]. Although direct evidence linking KRT76 to fetal outcomes in aneuploid pregnancies remains limited, animal studies highlight its importance: KRT6 deficiency has been shown to induce inflammation and early embryonic lethality, underscoring its role in epithelial protection and fetal viability [23,24,25,26].

In contrast, several structural and cytoskeletal proteins, including keratin 31 (KRT31), keratin 16 (KRT16), and lamin A/C (LMNA), were found less abundant in the aneuploid pregnancy group, suggesting altered placental cell integrity, trophoblast differentiation, and stress responses [27,28,29]. Cell adhesion molecules, including protocadherin 12 (PCDH12), nectin-3 (NECTIN3), and cadherin-related family member 5 (CDHR5) were also less abundant within the aneuploid pregnancy group, indicating disrupted intercellular junctions and placental morphogenesis. PCDH12, in particular, has been shown to play a pivotal role in endothelial–trophoblast interaction and vascular remodeling [30,31], while cadherins and nectin dysregulation has been associated with fetal growth restriction and pre-eclampsia [32].

**Extracellular Matrix & Vascular Remodeling.** Within this study, lower urinary abundance of extracellular matrix proteins such as matrix remodeling-associated 8 (MXRA8) and anthrax toxin receptor 1 (ANTXR1) were observed in aneuploid pregnancies. Both proteins are known to regulate endothelial differentiation and angiogenesis, and their reduced levels may indicate impaired vascular remodeling as a process closely linked to pre-eclampsia, fetal growth restriction, and preterm birth [33,34].

Von Willebrand factor A domain-containing protein 1 (VWA1), structurally related to von Willebrand factor (VWF), was detected at higher abundance in the aneuploid group. Although its precise role in pregnancy and placental function remains incompletely understood, VWA1 is expressed in placental tissue. Altered levels of VWF have been reported in maternal plasma from trisomy 21 pregnancies [35], but this study represents, to our knowledge, the first observation of VWA1 changes in the urinary proteome associated with fetal aneuploidy.

Fibulin-1 (FBLN1), an extracellular matrix glycoprotein expressed in the placenta and embryonic tissues, was also found at higher abundance in the aneuploid group. FBLN1 is known to regulate cardiac, skeletal, and neuronal differentiation and its dysregulation has been linked to congenital heart defects [36,37], commonly observed in aneuploid pregnancies. Also, elevated plasma levels of FBLN1 have been reported in severe pre-eclampsia [38]. Beyond pregnancy, FBLN1 has been proposed as a biomarker of vascular and tissue remodeling in renal disease and cancer [39,40,41].

Fibrinogen alpha chain (FGA) is essential for coagulation and placental attachment, supports early embryonic development, with deficiency leading to placental instability and pregnancy loss [42]. While fibrinogen levels increase physiologically during pregnancy and are altered in several complications [43], urinary FGA changes in aneuploidy have not been previously described. In the present study, higher urinary abundance of FGA in aneuploid pregnancies aligns with previous proteomic evidence linking fibrinogen o abnormal placentation and chromosomal abnormalities [44], supporting its potential biomarker value.

**Metabolic & Epigenetic Regulators.** Urinary proteomic analysis revealed distinct alterations in metabolic and transcriptional regulators in aneuploid pregnancies. Alpha-2-glycoprotein 1 (AZGP1) and methionine adenosyltransferase 1 (MAT1) were found at higher abundance in the aneuploid pregnancy group, suggesting disturbances in lipid metabolism and methylation processes. AZGP1, a zinc-binding glycoprotein involved in lipid metabolism and immune modulation, increases physiologically throughout gestation [45], but its role in aneuploidy remains unclear. On the other hand, MAT1 has a central role in methylation and placental function and has been linked to metabolic imbalance and adverse outcomes in cases of MAT1A deficiency [46,47]. Together, these findings indicate metabolic and epigenetic perturbations potentially reflecting altered maternal–fetal communication.

Conversely, lower urinary abundance of several glycolytic enzymes (PGK1, PGK2, LDHB, GAPDH, ALDOB) and detoxification proteins (GSTM2, CRYM) suggests impaired energy metabolism, redox balance, and nutrient regulation in aneuploid pregnancies. Such changes may reflect shared mechanisms of placental dysfunction and maladaptive metabolic reprogramming. Among the less abundant proteins, mediator complex subunit 21 (MED21), choriogonadotropin subunit beta variant 1 (CGB1), and glycoprotein hormones alpha chain (CGA) highlights potential disturbances in transcriptional and epigenetic regulation. MED21, a core subunit of the mediator complex acts as a central hub for transcriptional regulation, but its role in placental biology or fetal aneuploidy remains unexplored. CGB1 and CGA, encoding subunits of chorionic gonadotropin suggest altered trophoblast differentiation and endocrine signaling, consistent with impaired hormonal regulation in aneuploid pregnancies.

**Immune Regulation, surveillance and coagulation factors**. Two immune regulatory molecules, CD300 and PVR (CD155) were found at higher abundance in the urine of women carrying aneuploid fetuses. Both proteins play key roles in modulating maternal–fetal immune tolerance: CD300 regulates innate and adaptive responses on myeloid and lymphoid cells, while PVR interacts with checkpoint receptors (DNAM-1, TIGIT, CD96) on NK and T cells to balance immune activation and tolerance. Altered expression of these molecules has been linked to antifetal rejection [48] and immune-mediated pregnancy disorders [49]. However, despite their recognized immunological roles, neither CD300 nor PVR has been established as a biomarker of fetal aneuploidy.

Conversely, several immune proteins and coagulation factors were detected at lower abundance in the aneuploid pregnancy group, including IGHG3, IGHV3-49, SERPINA5, SERPINA4, HRG, and KLK1. Reduced immunoglobulins may reflect altered antibody transfer or immune dysregulation at the maternal–fetal interface [50,51,52]. Similarly, decreased levels of two serine protease inhibitors, SERPINA5 (protein C inhibitor) and SERPINA4 (kallistatin) have been associated with recurrent miscarriage [53], and pre-eclampsia [54]. While these specific serpins have not been directly linked to fetal aneuploidy, other family members, such as SERPINA3 and SERPINA1, have been reported as dysregulated in Down and Edwards syndromes [13,55]. In addition, lower urinary levels of histidine-rich glycoprotein (HRG) and kallikrein-1 (KLK1) suggest impaired angiogenesis and kallikrein–kinin signaling, processes implicated in pre-eclampsia and intrauterine growth restriction [56,57,58].

**Maternal–Fetal Interface Signaling and Communication Proteins.** In this study, several signaling and regulatory proteins involved in placental development, trophoblast invasion, extracellular matrix remodeling, and immune regulation were detected at lower urinary abundance in aneuploid pregnancies, including YWHAZ, EPHB3, CTSV and SLC3A2. YWHAZ (14-3-3ζ), a multifunctional adaptor protein regulating cell cycle, apoptosis, and signaling, has been implicated in placental pathology [59] and cancer biology [60]. Dysregulation of the miR-30c-5p/YWHAZ axis has been linked to vascular and inflammatory disturbances in intrauterine growth restriction (IUGR) and pre-eclampsia [59] suggesting that reduced YWHAZ may similarly reflect altered placental signaling in aneuploid pregnancies. Similarly, EPHB3, a receptor tyrosine kinase of the ephrin family, was also less abundant in our aneuploid pregnancy group. Literature evidence indicates that ephrin–EPHB signaling regulates cell adhesion, migration, and vascular remodeling in the placenta and its disruption can impair trophoblast invasion and contribute to pre-eclampsia [61].

We also observed reduced cathepsin V (CTSV), a lysosomal protease normally expressed in extravillous trophoblasts during myometrial invasion. This finding further supports impaired trophoblast invasion and abnormal placental adherence; opposite to the upregulation reported in placenta accreta spectrum (PAS) disorders, where excessive trophoblast invasion occurs [62].

Finally, SLC3A2 (4F2 heavy chain/CD98hc), a placental amino acid transporter regulating nutrient exchange and integrin signaling, was decreased in our dataset. Similar reductions have been described in pregnancies complicated by fetal growth restriction and pre-eclampsia [63,64].

**Chaperones and Stress Response.** In this study, HSPA2 (heat shock-related 70 kDa protein 2)—a member of the HSP70 family involved in protein folding, cellular stress regulation, and maternal immune modulation [65][]—was detected at lower urinary abundance in aneuploid pregnancies. This observation suggests enhanced placental stress, consistent with prior reports linking HSP70 dysregulation to pre-eclampsia and fetal growth restriction. However, urinary patterns remain inconsistent, emphasizing the need for validation in larger cohorts.

Overall, the urinary proteomic alterations observed in this study reflect disturbances in immune–metabolic balance at the maternal–fetal interface, aligning with previous evidence that chromosomal abnormalities disrupt immune signaling and metabolic pathways during gestation [66,67].

**Comparison with a reference urinary proteome**. To gain a better perspective on matrix specificity, we compared the urinary proteome identified in this study with a comprehensive normal urinary proteome reported by Zhao et al. [17] derived from 24 healthy adults (12 males, 12 females; mean age 38  ±  11 years). Notably, several differences need to be contextualized in relation to the referenced study: differences in sample demographics, preparation workflows, and analytical instrumentation may therefore partly account for variability in protein detection between studies.

Of the 861 proteins identified, 698 (81.1%) overlapped with the normal urinary proteome reported by Zhao et al. (Figure 1, Table S1). This substantial overlap confirms methodological robustness while also highlighting a distinct subset of proteins that may be specific to pregnancy and fetal aneuploidy. Functional annotation highlighted proteins involved in binding, catalysis, regulation, metabolism, and development, consistent with the immunologically active and metabolically adaptive state of pregnancy [68,69].

Among the 163 uniquely detected proteins, nine of them (SERPINA5, PLG, COL18A1, FOLR1, KLK11, MSLN, MMP7, GSTM5, RNASE2) were linked to extracellular matrix remodeling, angiogenesis, oxidative stress, and coagulation regulation—core processes in placental adaptation and maternal–fetal signaling. Several of these proteins have been previously implicated in placental dysfunction and chromosomal abnormalities. For example, collagen dysregulation and oxidative stress were documented in Down syndrome placentas [70], while FOLR1 was reported essential for folate transport and fetal neural development, linking it to altered folate metabolism in aneuploidy. Similarly, SERPINA5 and PLG mediate coagulation and fibrinolysis, while GSTM5 participates in oxidative stress defense—mechanisms often disturbed in chromosomal abnormalities and reported in trisomy 21 placentas [71].

**Study Limitations and Future Directions.** Several limitations of the present study warrant recognition. First, this study included a small cohort that limits the statistical power and generalizability of our results. Considering the small exploratory cohorts such as the present study, stringent FDR correction (e.g., Benjamini–Hochberg at 0.05) can be overly conservative, eliminating all potentially meaningful features and thereby masking biologically relevant signals. Consequently, unadjusted *p*-values (*p* ≤ 0.05) and fold-change thresholds (|fold change| ≥ 1.2) were applied to identify exploratory trends rather than definitive biomarkers. Future studies should include larger, prospective cohorts to address inter-individual variability and enhance robustness. While this exploratory analysis identified 42 differentially abundant proteins distinguishing aneuploid from euploid pregnancies, these findings should be interpreted as preliminary.

Specificity represents an additional limitation. The urinary proteome reflects a mixture of maternal and fetal physiological processes and cannot be unambiguously attributed to fetal origin. Currently, no proteins can be unambiguously attributed to fetal origin, and observed changes may also be influenced by maternal metabolic, immunological, or inflammatory states. Observed changes may also be influenced by maternal metabolic, immunological, or inflammatory states. Although several differentially abundant proteins (DAPs) identified in maternal urine have been reported in other matrices such as plasma or serum, their expression is not uniquely linked to fetal aneuploidy. Nevertheless, urine offers a unique, non-invasive window into fetal–maternal communication and the proteomic signatures detected may still provide biologically meaningful insights.

Overall, this exploratory study highlights several biologically relevant urinary proteins associated with immune regulation, coagulation, metabolism, extracellular matrix remodeling, and transcriptional control. These findings provide a foundation for future research aimed at validating candidate biomarkers and elucidating the mechanisms of maternal–fetal signaling reflected in the urinary proteome.

## 5. Conclusions

This exploratory study provides initial insights into the maternal urinary proteome associated with fetal aneuploidy. Using a label-free, mass spectrometry-based approach, we identified distinct protein abundance patterns involving immune regulation, metabolism, extracellular matrix remodeling, and epithelial barrier function suggesting disrupted maternal–fetal metabolic communication and placental adaptation in aneuploid pregnancies. While preliminary, the findings underscore the potential of maternal urine as a non-invasive matrix for investigating pregnancy-associated molecular changes and lay the groundwork for future studies aimed at biomarker validation and mechanistic exploration.

## Figures and Tables

**Table 1:** Demographic and clinical characteristics of the study participants.

Characteristics	Case Group (n_1_ = 9) *	Control Group (n_2_ = 15) *	*p*-Value
Maternal age (years)	38.6 (31.3–40.3) {26.4–43.8}	29.4 (26.5–31.9) {15.8–37.6}	0.007
Gestational age (weeks)	16.7 (16.3–17.1) {15.6–17.6}	12.4 (12.1–12.6) {12–13.1}	<0.001
Marital status Married	5 (55.6)	12(80.0)	0.356
Place of origin Rural	3 (33.3)	4 (26.7)	1.0
Educational Level Low	7 (70)	8 (53.3)	0.678
Smoking Status Smoker	4 (44.4)	1 (6.7)	0.047
Dysmenorrhea	1 (10)	-	0.40
Threatened miscarriage	1 (10)	2 (13.3)	1.0
Folic acid supplementation	8 (88.8)	15 (100)	0.375

Quantitative data is presented as median (quartile 1–quartile 3) {minimum–maximum} and categorical data is reported as absolute (relative, %) frequency; * complete case data for VG in control group = 14, for height in case group = 8, for dysmenorrhea and educational level in case group = 10.

## Data Availability

The complete dataset, including raw data and search outputs, has been deposited in the MassIVE public repository (MSV000099309; doi:10.25345/C57H1F09Z).

## Supplementary Materials

The following supporting information can be downloaded at: https://www.mdpi.com/article/10.3390/cimb47120973/s1.
