# Supplementary material for: Exploratory Urinary Proteomic Profiling in Pregnancies with Fetal Aneuploidies: Molecular Insights into Maternal–Fetal Metabolic Communication
Source: Curr Issues Mol Biol. 2025 Nov 24;47(12):973. doi: 10.3390/cimb47120973 (PMC12731864; doi:10.3390/cimb47120973)
Supplement: Supplementary file 1 [file cimb-47-00973-s001.zip › cimb-3974261-supplementary.pdf]

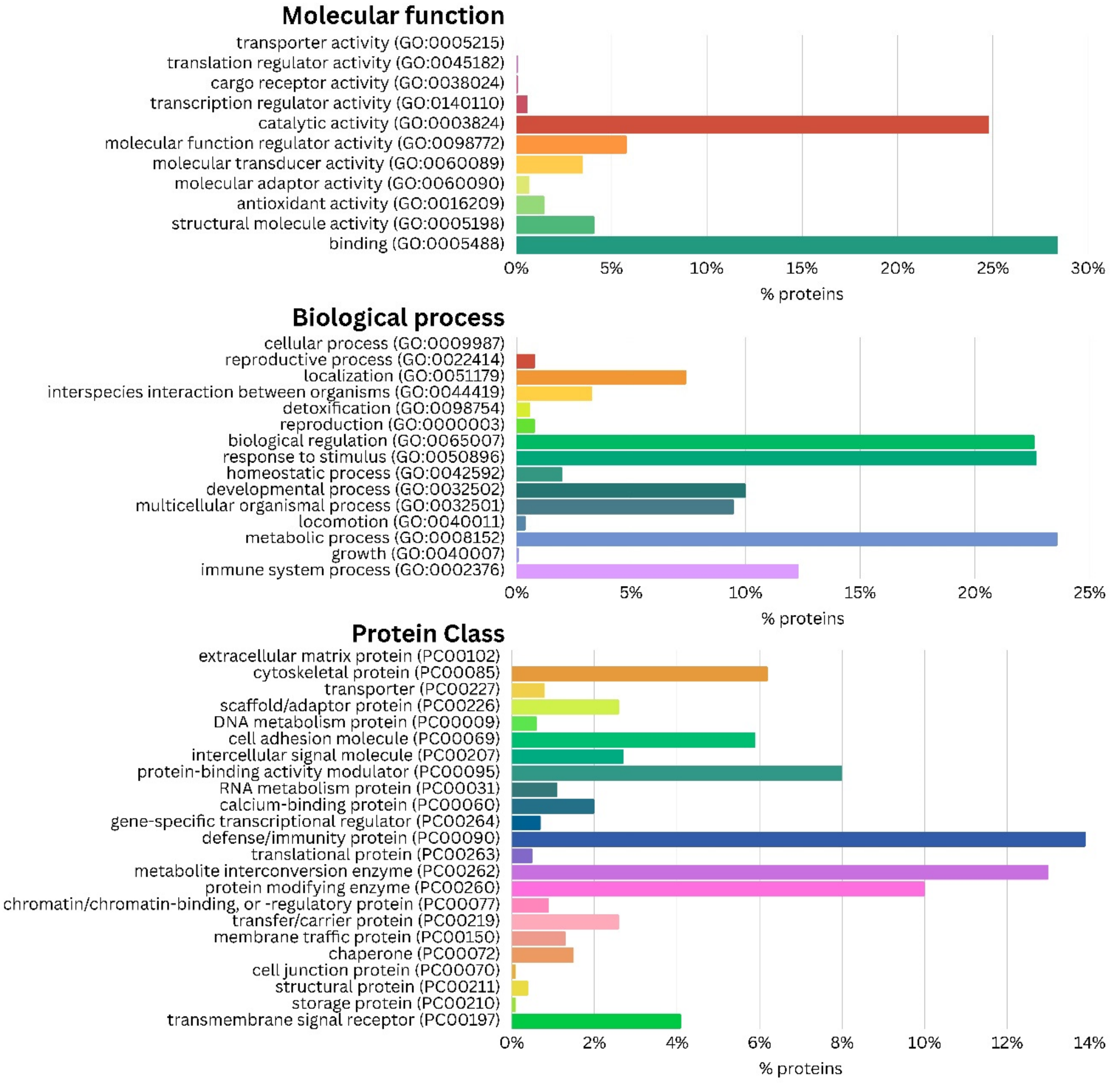

Figure S1. Functional annotation of the maternal urinary proteome using the PANTHER Classification System (http://www.pantherdb.org) and GO annotations: Molecular function, biological process and Protein Class.

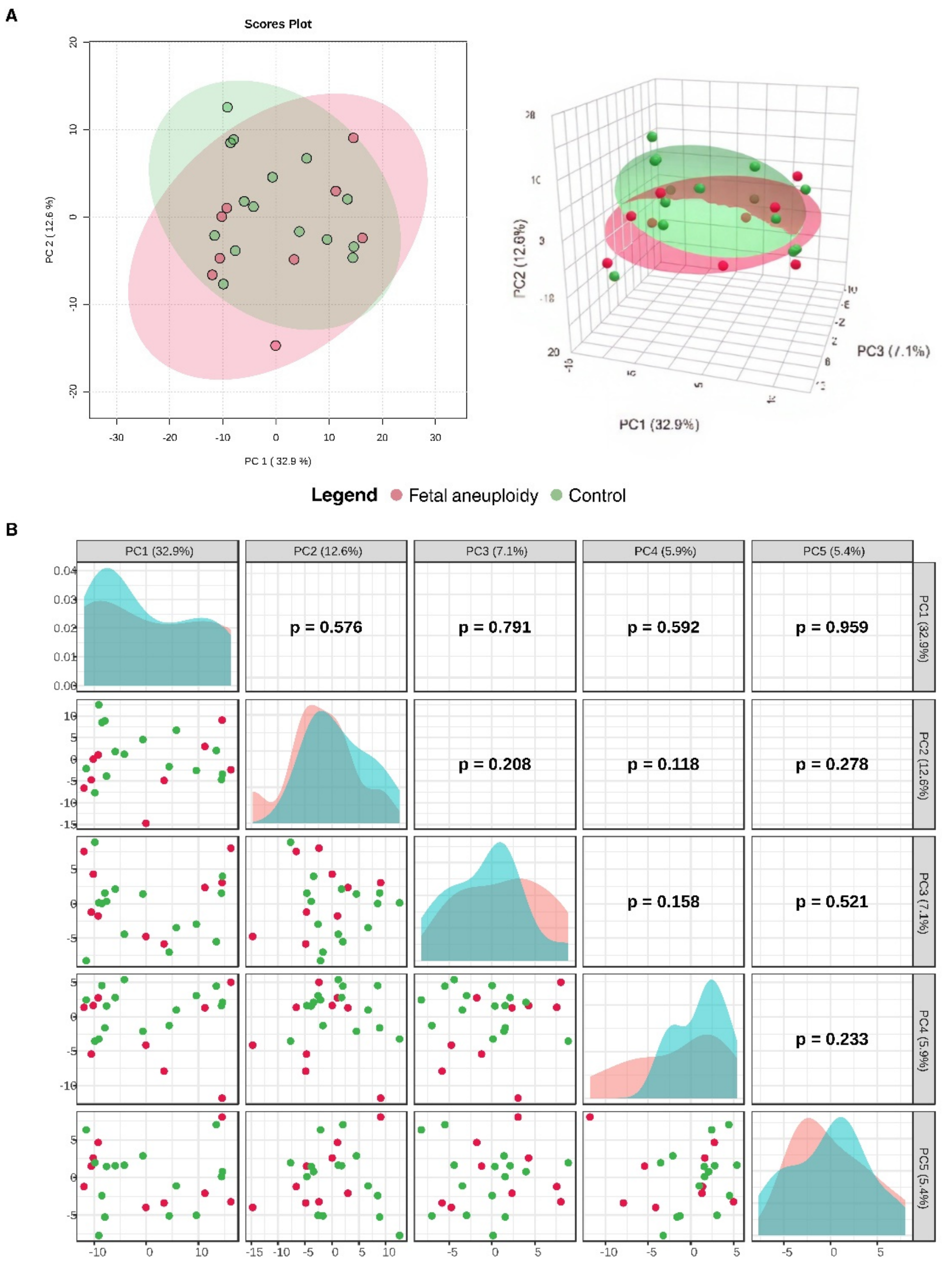

Figure S2. Principal Component Analysis (PCA) of proteomic profiles. (A) Global PCA illustrates the variance structure between study groups. Left: 2D PCA scores plot of the first two principal components (PCs), with the percentage of explained variance indicated in brackets. Right: 3D PCA plot of the first three PCs. (B) Pairwise score plots of selected principal components.

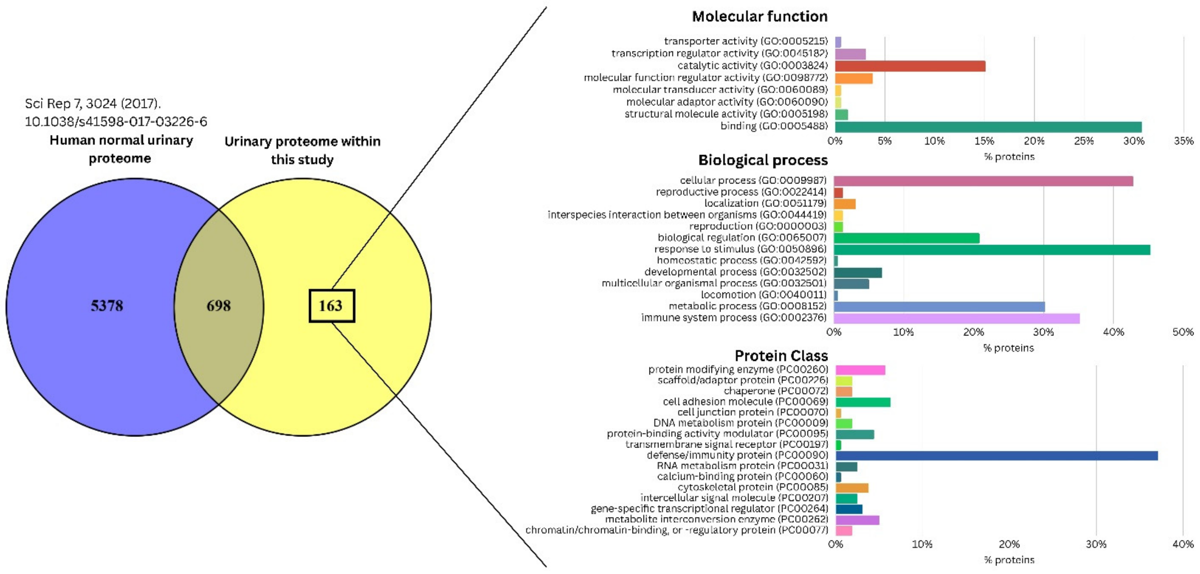

Figure S3. Functional annotation of the proteins uniquely identified within this study. Left panel: Venn diagram illustrating the overlap between the urinary proteome identified in this study and the normal human urinary proteome reported by Zhao et al. [17]. Right panel: Functional annotation of the proteins uniquely identified within this study using the PANTHER Classification System (http://www.pantherdb.org) and GO annotations: Molecular function, biological process and Protein Class.
